# Open Sn Framework Structure Hosting Bi Guest atoms–Synthesis, Crystal and Electronic Structure of Na_13_Sn_26_Bi

**DOI:** 10.1002/chem.202403592

**Published:** 2024-11-27

**Authors:** S. Zeitz, M. Boyko, S. Ponou, V. Hlukhyy, T. F. Fässler

**Affiliations:** ^1^ School of Natural Science Technical University of Munich, Chair of Inorganic Chemistry with Focus on Novel Materials Lichtenbergstraße 4 D-85747 Garching Germany

**Keywords:** Zintl phases, Electronic structure, Bismuth

## Abstract

The large variety of structures of Zintl phases are generally well understood since their anionic substructures follow bonding rules according to the valence concept. But there are also exceptions, which make the semiconductors especially interesting in terms of structure‐property relationships. Although several Na‐Sn‐Pnictides with a variety of structural motives are known, up to this point no ternary compound in the Na‐Sn‐Bi system has been described. In this paper we present the Zintl‐phase Na_13_Sn_25.73_Bi_1.27_ comprising a complex, open‐framework structure of Sn atoms, with one mixed Sn/Bi site, hosting Na atoms. An additional Bi atom is loosely connected with only weak contacts to the framework filling a larger cavity within the network. According to band structure calculations of the two ordered variants with either full occupation of the mixed site with Sn or Bi, resulting in Na_13_Sn_26_Bi and Na_13_Sn_24_Bi_3_, respectively, both compounds are semiconductors with band gaps of 0.5 eV. A comparison of the band structures with the structurally related binary compounds Na_5_Sn_13_ and Na_7_Sn_12_ shows that only the perfectly charge balanced Na_7_Sn_12_ is a semiconductor whereas Na_5_Sn_13_ is metallic. The rather specific electronic situation in the ternary compound is traced back to the loosely bound Bi atom, which acts as a guest atom according to Bi_x_@Na_13_Sn_26‐y_Bi_y_, with x=1 and y=0.27, capable to change its oxidation state and thus to uptake additional electrons allowing the system to be a semiconductor. Therefore, Na_13_Sn_25.73_Bi_1.27_ can be understood as a rare example of an open framework structure of Sn atoms comprising Bi atoms in the cavities.

## Graphical abstract

Na_13_Sn_26‐x_Bi_1+x_ is the first compound in the Na‐Sn‐Bi ternary system. Sn atoms form a three‐dimensional open framework hosting Na and also one loosely bound Bi guest atom. Interestingly, for both x=0 and x=2 results a semiconducting small indirect band gap Zintl compound owning the same atomic structure but different electron numbers.

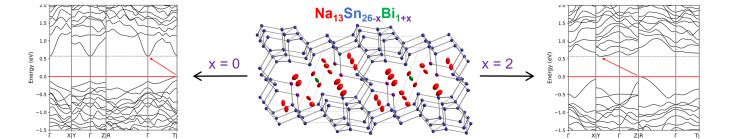

## Introduction

Zintl phases are a versatile compound class since this subgroup of intermetallic compounds allows for a deeper understanding of structure property relationships. The structures can be understood by the formal electron transfer from the electropositive metal component to the more electronegative p‐block metals. Subsequently, the anionic substructure can be determined by evaluation the valency of each element according to the (8‐N) rule. Since Zintl phases are in general semiconducting, they are especially prominent materials allowing for band gap tuning which is an important property for materials with thermoelectric or optoelectronic applications. Variation by atom‐to‐atom substitution in binary compounds such as *A*_n_ *E* ^N^_m_ with *A*=alkali metal and *E* ^N^ element of main group N that correspond to electron‐precise Zintl phases or that are rather close to an electron‐precise count are of particular interest. The atoms of the polyanion adapt the 8‐N rule and the addition of a small amount of a main‐group elements *E* ^N−1^ or *E* ^N+1^ could lead to changes in the size and type of the band gap, trigger metal to insulator (semiconductor) transitions, or induce structural changes or combinations of those.[^1^, ^2^]

**Figure 1:**
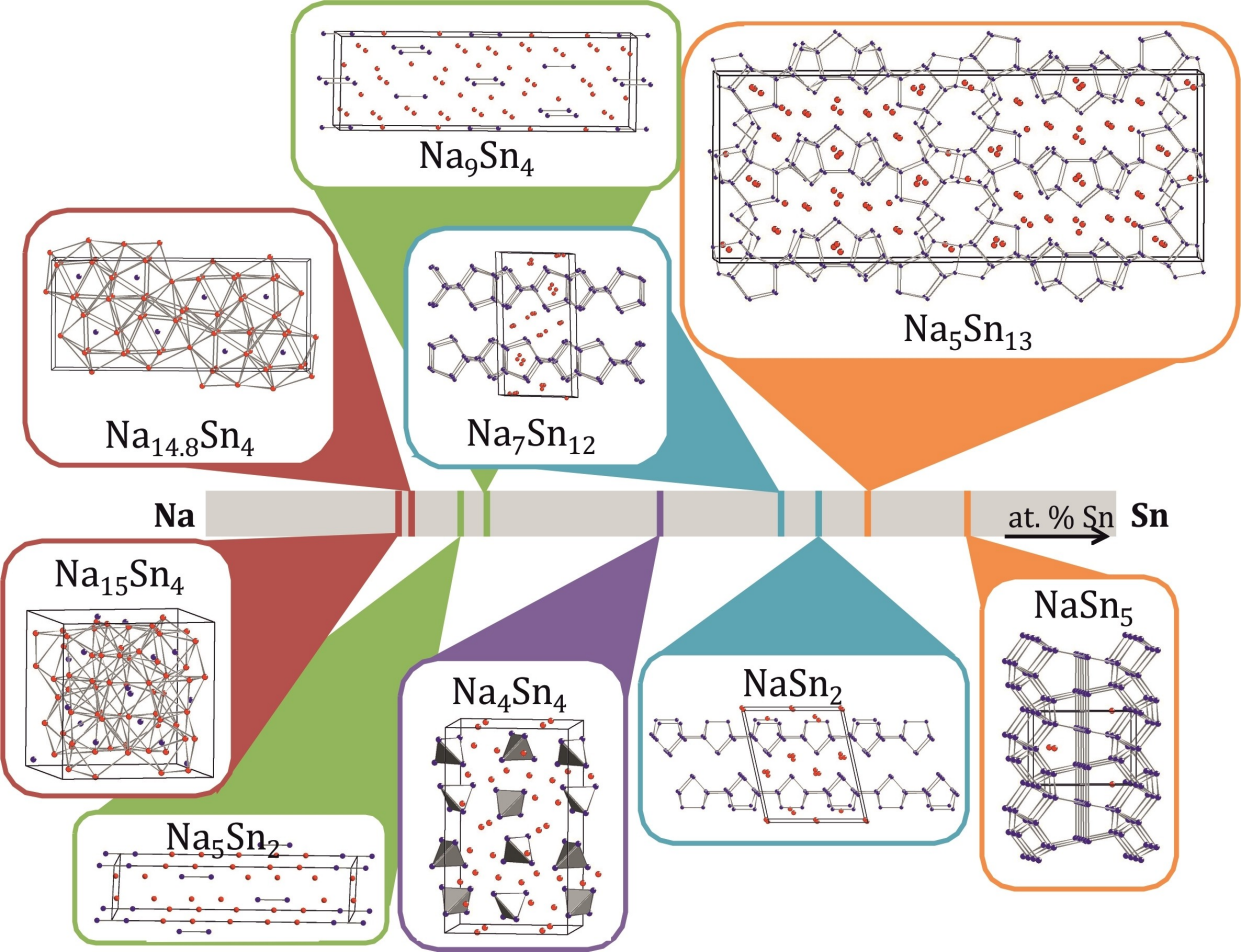
Fragments of the crystal structure of the binary phases in Na−Sn binary system in dependency of the Na : Sn ratio.^[3]^ Color code according to dimensionality of the Sn anionic frameworks: isolated Sn atoms–red rectangles; dimeric {Sn_2_} units–olive green; two‐dimensional polyanions–aqua blue; three‐dimensional Sn‐network–orange; isolated clusters–purple.

The systematic study of binary phase diagrams such as the Na−Sn system has revealed interesting trends in the relationship between the atomic and electronic structure of Na−Sn intermetallic compounds. Merely changing the Na : Sn ratio leads to a wide variety of Sn frameworks, including discrete, isolated Sn atoms in Na_15_Sn_4_ and Na_14.8_Sn_4_; dimeric {Sn_2_} units in Na_5_Sn_2_ and Na_9_Sn_4_; two‐dimensional polyanions in Na_7_Sn_12_ and NaSn_2_; three‐dimensional Sn‐network in Na_5_Sn_13_ and NaSn_5,_ as well as isolated tetrahedral cluster {Sn_4_}^4−^ in NaSn (Na_4_Sn_4_) as depicted in Figure 1.[^3^, ^4^, ^5^, ^6^, ^7^, ^8^, ^9^, ^10^, ^11^, ^12^]

We investigate here the influence of the electron count on the structure by aliovalent substitution of Sn in binary Zintl phases of the Na−Sn system while trying to dope them with a relatively small amount of Bi. As a starting point we used the compound Na_5_Sn_13_, which has been regarded as a Zintl phase.^[11]^ However in order to reach the necessary number of bonds according to the 8‐N rule, *Corbett* regarded a very long Sn−Sn contact of 3.6  Å as a two‐electron‐two center bond. Neglecting a bonding contact consequently leads to the formation of two lone pairs at the Sn atom which requires, for a charge‐balanced compound, a higher negative charge at both Sn atoms. In consequence, the reported Na_5_Sn_13_ would correspond to an electron‐deficient Zintl phase. However, the postulation of such a long covalently bonding contact is rather vague. Substituting an appropriate number Sn atoms by Bi lead in other cases to a stabilization of electron deficient compounds. For example, corresponds the chiral clathrate K_6+x_Sn_25_ to an electron‐deficient phase. Stabilization by Bi resulted in the formation of the electron‐precise Zintl phase K_6_Sn_23_Bi_2_.^[13]^ Similarly leads the Bi versus Pb substitution in the in the Laves phase KPb_2_ to a metal to semiconductor transition, by lowering the symmetry with a Peierls like distortion of the Bi/Pb network.^[1]^

Interestingly, no ternary Na‐Sn‐Bi phases are reported yet, whereas for the lighter homologues of Bi, seven compounds in the Na‐Sn‐*Pn* systems are reported (*Pn*=P, As and Sb), which show intriguing structure motifs reaching from one‐ to three‐dimensional networks (Supporting Information Figure S1). The compounds similarly appear, mostly, as electron‐precise Zintl phases with localized bonds, however, also compounds in which the bond description is less clear are known. In Na_10_Sn_2_ *Pn*_6_[^14^, ^15^] (*Pn*=P, As) edge‐sharing Sn*Pn*_4_ tetrahedra form dimeric {Sn_2_ *Pn*_6_}^10−^ anions that are isolated by Na atoms. Na_2_SnAs_2_ and Na_5_SnSb_3_ possess three‐dimensional structures with vertex‐sharing Sn*Pn*_4_ tetrahedra and Na_2_SnAs_2_ ^[16]^ comprises tetrahedrally coordinated Sn atoms with {Sn_4_As_10_} adamantane‐like units. In Na_5_SnSb_3_ ^[17]^ the main structural element can be described as SnSb_4_ tetrahedra forming zig‐zag chains. NaSnP^[18]^ as well as NaSn_2_As_2_ ^[19]^ feature both two‐dimensional layers, similar to As layers in the structure of grey arsenic. However, NaSnP corresponds to an electron‐precise Zintl phase according to Na^+^, (3b‐Sn)^−^ and 3b‐As (3b=three‐fold bonded), and interestingly is NaSn_2_As_2_ with exclusively 3b‐Sn and 3b‐As atoms an electron‐deficient Zintl phase unless very long contacts between two Sn atoms with distances of 3.3 Å are anticipated as covalent bonds.

We report here on our approach to electronically modify Na_5_Sn_13_ featuring an anionic network where Sn atoms are 2‐, 3‐ and 4‐fold bonded by doping with more electron rich Bi atoms. We find the formation of a compound with a closely related framework. Surprisingly, Bi atoms do not only partially substitute Sn atoms as known for other compounds, but also act as loosely bounded host atoms within the Sn network. multicenter bonding Na_12.73_Sn_25.73_Bi_1.27_.[^11^, ^20^, ^21^]

## Results and Discussion

### Synthesis and Characterization

The compound Na_13_Sn_25.73_Bi_1.27_ was synthesised through a high temperature reaction from a mixture of elements Na : Sn:Bi with the composition 5 : 12 : 1. The microcrystalline product contained some unreacted Sn as a side phase (Figure S2 Supp. Inform.) as well as some reflections, that could not be assigned to a known phase. Attempts to synthesize the title compound as phase pure material were not successful. A dark, almost black in color block‐shaped single crystal was isolated from the reaction product of a sample synthesized with a longer dwelling time at 270 °C. For details see below.

### Crystal Structure

Single crystals show triclinic symmetry and the refinement of the single crystal diffraction data result in the crystallographically determined composition Na_13_Sn_25.73(2)_Bi_1.27(2)_. All parameters of the single crystal refinement can be found in Table 1. The atomic coordinates for twenty‐one crystallographic independent atoms positions are listed in Table S1 together with anisotropic displacement parameters for all atoms given in Table S2 (Supporting Information). High residual electron density at the Sn2 position suggested the formation of a statistical mixture of Sn and Bi. The refinement showed a Sn/Bi ratio of 0.861/0.139(7) on position *E*2. No correlation between the occupancy of Na positions and a possible partial occupancy of the *E*2 site was observed revealing no significant defects on any Na position. Another disorder was found for the Bi1 position, which was refined with two split positions (Wyckoff site 2i) with an occupancy of 50 % each.

**Table 1:** Crystallographic data and data and selected details of structure refinement for the compound Na_13_Sn_25.73(2)_Bi_1.27(2)_.

Formula	Na13Sn25.73(2)Bi1.27(2)
Formula weight (g⋅mol^−1^)	3618.17
Space group	*P* $\bar{1}$ (No. 2)
*Z*	1
Unit cell parameters (Å)	*a=*9.0826(4) *b=*11.2527(5) *c=*13.2278(6) *α=*112.114(4)° *β=*99.818(4)° *γ=*101.379(4)°
Volume (Å^3^)	1182.4(1)
*D*_calcd._ (g⋅cm^−3^)	5.081
Abs. coeff. (mm^−1^)	18.128
*F*(000) (e)	1535
Crystal shape/color	block/black
Temperature (K)	150
*Θ* range (deg)	3.038–27.498
Range in *hkl*	±11±14±17
Reflections collected	42179 (*R*_ϭ_=0.0216)
Unique reflections	5410 (*R*_int_=0.0305)
Data/parameter	5410/189
GOF on F2	1.211
*R_1_, wR_2_ (I>2 ϭ(I))*	0.0355, 0.0844
*R_1_, wR_2_ (all data)*	0.0458, 0.0870
Largest diff. peak/hole (e Å^−3^)	2.585 and‐1.944

**Figure 2:**
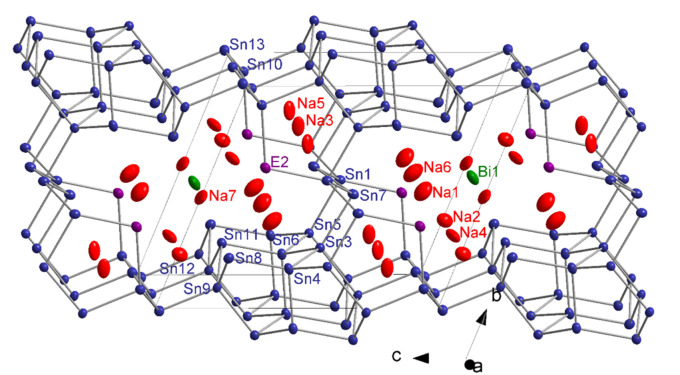
Extended unit cell of the compound Na_13_Sn_25.73_Bi_1.27_. The three‐dimensional framework is shown on an extended part of the unit cell. Na, Sn, Bi and the mixed Sn/Bi atoms are shown in red, blue, green, and violet color, respectively. Thermal ellipsoids are drawn with 90 % probability level.

Na_13‐*x*_Sn_26‐*x*_Bi_1+*x*_ is the first and only representative of the Na‐Sn‐Bi ternary system. Its structure, shown in Figure 2, with all atoms shown as ellipsoids with 90 % probability. All atomic positions, isotropic and anisotropic displacement parameters can be found in Table S1 and S2 in the Supporting information.

**Figure 3:**
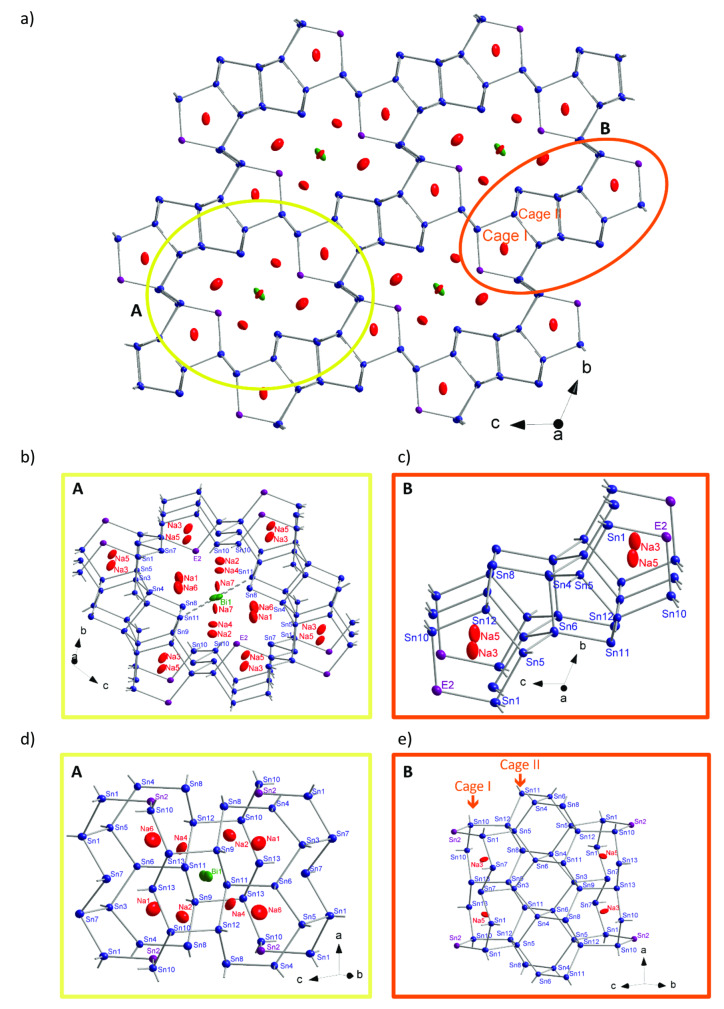
Structure of the polyanionic Sn network of Na_13_Sn_25.73_Bi_1.27_, a) featuring two types of structural fragments: fragment A and B shown by the yellow and orange circle, respectively. b) enlargement of A showing cage III hosting atom B1, c) enlargement of B featuring the network of Sn pentagons that forming the cages I and II. a)–c) shown as a projection along the a direction. d) and e) show the direction perpendicular to a with emphasizing the cages. Sn, Bi and Sb/Bi atoms are shown with blue, green, and violet color. Displacement ellipsoids are shown at 90 % probability.

Twelve tin atoms (position Sn1, Sn3‐Sn13) together with a position that possess a Sn/Bi statistical mixture (*E*2) form a complex three‐dimensional network (Figure 2 and Figure 3) which is related to the binary phase Na_5_Sn_13_.^[11]^ The network contains two major motifs that are denoted as fragments **A** and **B** shown in detail in Figure 3b and 3c, respectively. Fragment **B** is composed by four fused cages ‐ two cages of type **I** and **II**, each, in the sequence **I–II**‐**II–I**. The cages form parallel channels along the *a* direction (Figure 3e). Four such fragments **A** enclose the larger cavity **B** that hosts the Bi1 atom. In Figure 3d and 3e the two fragments **A** and **B** are shown along the *a* axis. Cages II and fragment **A** are encapsulating Na atoms as shown in Figure 3: Na3 and Na5 are located inside of cage **I**, whereas all the remaining Na atoms (Na1, Na2, Na4, Na5, Na6 and Na7) are located inside fragment **A**. All atoms Sn1, Sn3 to 13 as well as all Na positions are fully occupied, while the Sn2/Bi2 position is occupied by 86.1 % Sn and 13.9 % Bi.

Homoatomic Sn−Sn distances within the open framework of Na_13_Sn_25.73_Bi_1.27_ are between 2.822 and 2.947 Å (Table 2), which suggests covalent interactions: Sn7, Sn8, Sn11 and Sn13 are three‐, Sn1, Sn3, Sn4, Sn5, Sn6, Sn9, Sn10 and Sn12–four‐fold‐bonded to neighboring atoms. The atoms of the statistical mixture Sn/Bi (*E*2) are two‐fold bonded with *d*_Sn1–*E*2_=2.965 Å and *d*_Sn10–*E*2_=2.985 Å.

**Table 2:** Selected interatomic distances in the compound Na_13_Sn_25.73_Bi_1.27_. For details see Table S4 (SI).

Atom types	Distance range(Å)
Sn‐Bi	3.145(8)–3.456(8)
Sn‐*E*	2.965(1)–2.985(1)
Sn‐Sn	2.822(1)–2.947(2)
Na‐Bi	3.22(3)–3.86(1)
Na‐*E*	3.243(7)–3.302(8)
Na‐Sn	3.186(8)–3.925(6)
Na‐Na	3.521(8)–4.080(9)

The coordination of the Bi1 atom, with a site occupancy of 50 %, differs since it comprises longer distances to its next neighbors: Distances to Sn11 are 3.145 Å and 3.455 Å, which are both significantly longer than the sum of the covalent radii of single‐bonded Bi (1.51 Å^[22]^) and Sn (1.40 Å^[22]^). Thus Na_13_Sn_25.73_Bi_1.27_ shows that Bi in Sn‐rich Na−Sn‐Bi compounds is either capable to substitute two‐bonded Sn atoms, preserving the integrity of the Sn network, or may act as a loosely bound guest atom within the framework.

**Figure 4:**
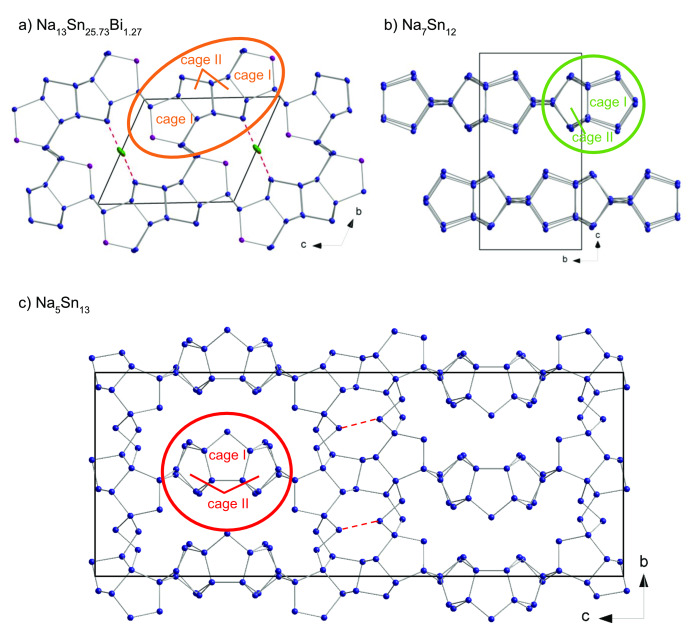
Structural relation of a) Na_13_Sn_25.73_Bi_1.27_ to the binary phases b) Na_7_Sn_12_ and c) Na_5_Sn_13_. In a) the elongated Sn−Bi interaction of 3.145 Å and for comparison in c) a non‐bonding Sn−Sn contact of 6.011 Å is shown as dashed line.

The structural relationship of Na_13_Sn_25.73_Bi_1.27_ to the binary phases Na_7_Sn_12_ ^[12]^ and Na_5_Sn_13_ ^[11]^ is shown in Figure 4. All three compounds contain type **I** and **II** cages that are constructed by Sn‐pentagons. In Na_13_Sn_25.73_Bi_1.27_ they are condensed to tetramers **I–II**‐**II–I** (orange ellipse in Figure 4a), forming a larger cavity as shown in Figure 3a (yellow ellipse). In Na_5_Sn_13_ similar cages appear as condensed trimers **II–I**‐**II** (red ellipse in Figure 4c), forming a three‐dimensional network together with additional fragments, whereas in Na_7_Sn_12_ dimers of the cages **I** and **II** are interconnected in *a*‐direction forming layers (green ellipse in Figure 4b).

### Electron Count

To get insight on the electron count of Na_13_Sn_25.73_Bi_1.27_ we investigated two boundary cases in which the mixed Sn2/Bi2 position is either fully occupied by Sn or by Bi. Full occupancy with Sn (case 1) results in the composition Na_13_Sn_26_Bi and the following electron count for the polyanionic covalent network: [(2b‐Sn^2−^)_2_(3b‐Sn^−^)_8_(4b‐Sn^0^)_16_] (nb = n‐fold bonded or n covalent bonds) leading to an overall charge of −12 for the three‐dimensional network. Since there are 13 Na ions for charge balance, consequently the 0b‐Bi1 guest atom should formally be considered as Bi^1−^ resulting in (Na^+^)_13_[(0b‐Bi^1−^)_1_(2b‐Sn^2−^)_2_(3b‐Sn^−^)_8_(4b‐Sn^0^)_16_]. Based on this consideration, Na_13_Sn_26_Bi is as a charge balanced Zintl phase. For the second case, with a composition of Na_13_Sn_24_Bi_3_, the atom connectivity is according to [(2b–Bi^−^)_2_(3b–Sn^−^)_8_(4b–Sn^0^)_16_] and a total charge of −10 results. With the same number of Na counter ions, the isolated Bi1 atom is assigned with a negative charge of −3 to remain charge balance and resulting in the sum formula (Na^+^)_13_[(0b–Bi^3−^)_1_(2b–Bi^−^)_2_(3b–Sn^−^)_8_(4b–Sn^0^)_16_]. According to the crystallographically determined composition obtained through the refinement of the SC‐XRD data, Na_13_Sn_25.73_Bi_1.27_ and the resulting ratio of (2b–Sn1)^2−^ and (2b‐Bi2)^−^ of 86 % to 14 % an average sum formula of (Na^+^)_13_[(0b–Bi^1−^)_0.861_(0b–Bi^3−^)_0.139_(2b–Bi^−^)_0.272_(2b–Sn^2−^)_1.728_(3b‐Sn^−^)_8_(4b‐Sn^0^)_16_] results and the guest atom 0b‐Bi1 takes up the required average surplus charge of 0.272 electrons remaining a charge balanced Zintl phase.

### Electronic Structure

The electronic structure of Na_13_Sn_25.73_Bi_1.27_ was calculated using two stoichiometrically precise model systems, Na_13_Sn_26_Bi and Na_13_Sn_24_Bi_3_, where the mixed Bi/Sn position was either fully occupied by Sn or Bi and the Bi1 position was fixed along the c direction in the ab‐plane, lowering its Wyckoff position from 2i to 1e.

**Figure 5:**
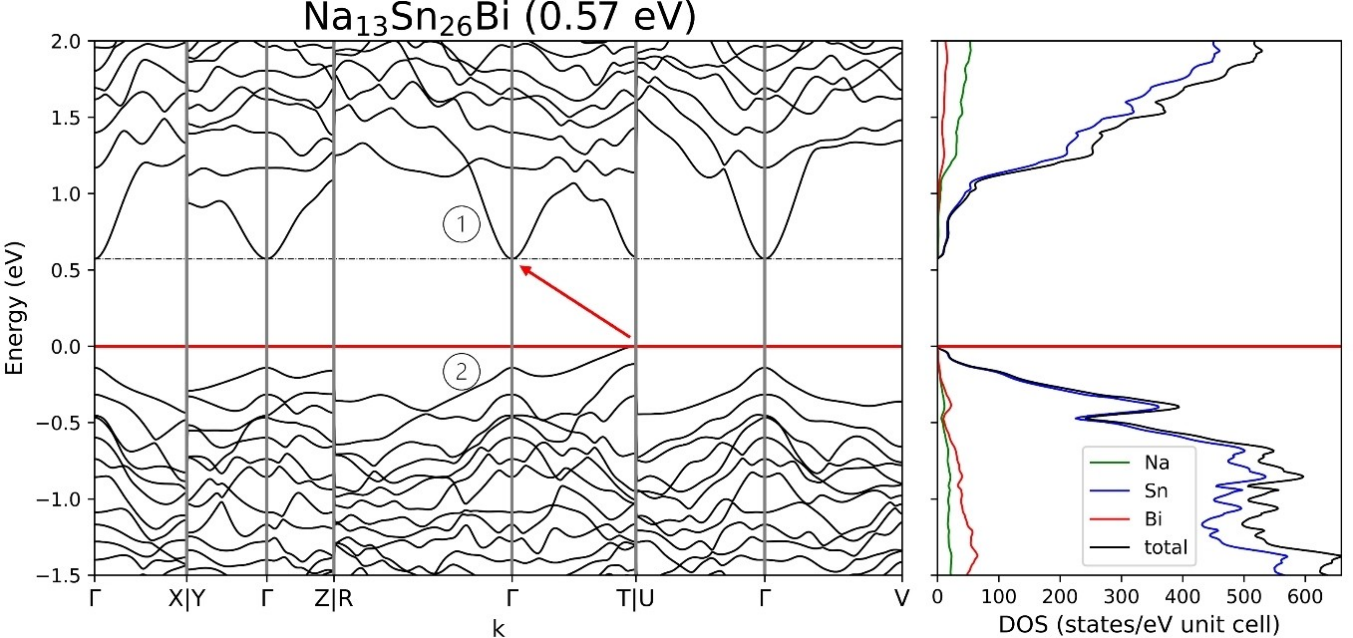
Band structure and density of states for Na_13_Sn_26_Bi with a band gap of 0.6 eV with the corresponding transition marked with an arrow.

**Figure 6:**
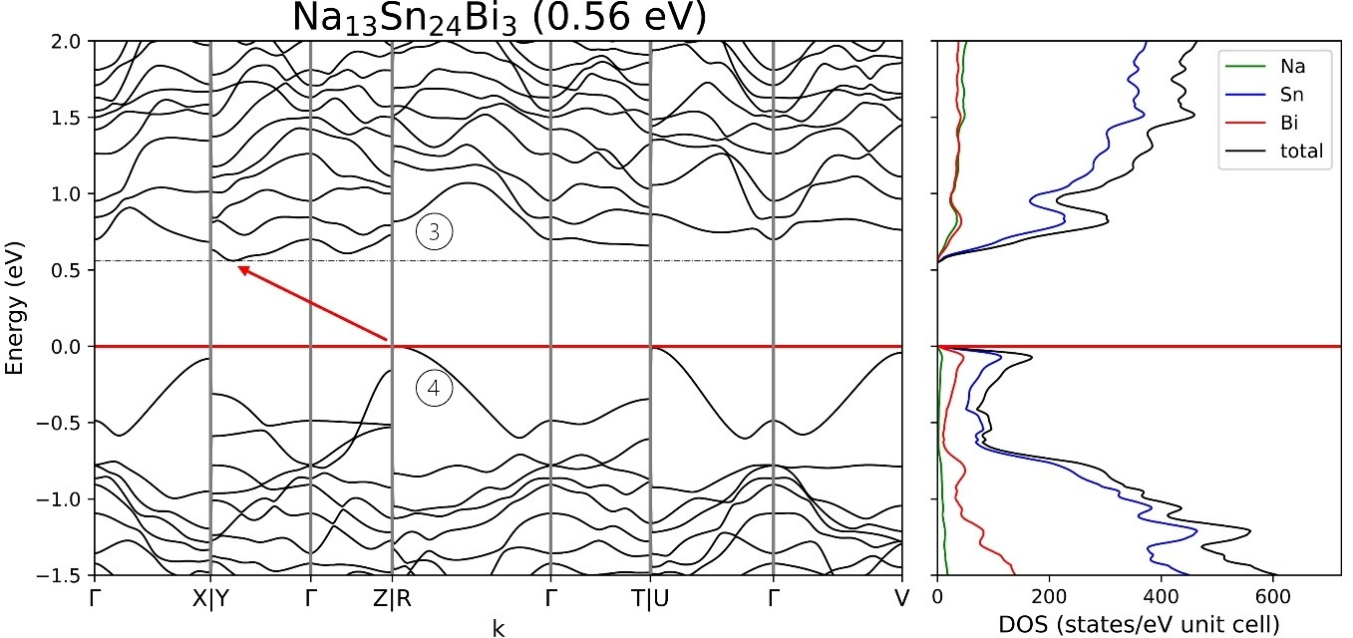
Band structure and density of states for Na_13_Sn_24_Bi_3_ with a band gap of 0.6 eV with the corresponding transition marked with an arrow.

The resulting band structures and density of states calculated with a DFT‐PBE0/TZVP level of theory (SVP for Na) are shown in Figure 5 and Figure 6, along lines between high symmetry points in the first Brillouin zone. Cell parameters and atom coordinates of the optimized structures coincide with the by SCXRD determined ones with a maximum deviation of 1.75 % (Table 7, SI). Since for both models one negative frequency of about −25 cm^−2^ was obtained, the structure was distorted and reoptimized in P1 symmetry. Here all atomic coordinates were refined without constraints. Interestingly changes the Bi1 atom, which was previously fixed on a special position, its coordinates by only a maximum of 2 %.

Both model compounds, Na_13_Sn_26_Bi and Na_13_Sn_24_Bi_3_, are indirect band gap semiconductors with band gaps of 0.6 eV, with a transition from T→Γ for Na_13_Sn_26_Bi and R→Y‐Γ for Na_13_Sn_24_Bi_3_. The band structures of the ordered models look in general similar but differ for the valence and conduction bands close to E_F_ with respect to the band dispersion and location of the valence band maximum (VBM) and conduction band minimum (CBM). For Na_13_Sn_26_Bi the band with the CBM has a dispersion of about 0.5 eV (①) while the band with the VBM is rather flat (②). For Na_13_Sn_24_Bi_3_ this is inverted, thus having a flat first conduction band (③) and a band with VBM (④) and a dispersion of 0.5 eV. Interestingly are the band gaps of both modelled compounds almost identical. The Na_13_Sn_24_Bi_3_ model possesses in comparison to Na_13_Sn_26_Bi in total 2 additional electrons. Considering a rigid band model to Na_13_Sn_26_Bi a shift of the Fermi‐Level to higher energy and filling the lowest conduction band is expected, transforming Na_13_Sn_24_Bi_3_ to a metal. However, applying the Bi richer model for the calculation, the conduction band of Na_13_Sn_26_Bi is not filled but rather in Na_13_Sn_24_Bi_3_ an additional band is added below the band gap, with a dispersion similar to the first conduction band in Na_13_Sn_26_Bi.

For both models the atom projected DOSs at the Fermi‐Level gives a more detailed insight on the electronic structure. In general, for both models Sn atoms have the highest contribution within the projected DOS around the Fermi‐Level followed by Bi and Na atom contributions. For the Na_13_Sn_26_Bi the contribution of Bi states in the top valence band is only minor while in Na_13_Sn_24_Bi_3_ they make up about one third of the total states and are especially present with a local maxim just below E_F_. There is no Bi contribution to the conduction bands of Na_13_Sn_24_Bi_3_ in contrast to a higher contribution of Bi states to the first conduction band of Na_13_Sn_26_Bi. Resolving the Bi states by their different atomic positions reveals that these states originate from the two bonded Bi2 position (see Figure 2, SI). This correlates with the chemical picture, that two‐bonded Bi atoms have a formal negative charge and two non‐bonding electron pairs (lone pairs) that energetically form the highest occupied orbitals.

Chemical bonding was further investigated by an analysis of the overlap population and the Mulliken charges (see Table S6–8, SI). Considering values of the overlap population larger than 0.2 as covalent bonds, the atom connectivity imposed by the electron count with 2b and 3b atoms in the framework as shown before can be confirmed. The overlap population between the isolated Bi1 and its closest Sn11 neighbour (Figure 3b) is 0.084 for Na_13_Sn_26_Bi and 0.094 for Na_13_Sn_24_Bi_3_, which accounts for a rather similar interaction for both models with the Sn11 position, but in both cases not in the range accounting for a single bond. The calculated Mulliken charges for Na_13_Sn_26_Bi are used to reflect the trend of the charge distribution. Notice, that Mulliken charges do neither correspond to ionic nor to formal charges but are capable to reflect trends within a structure. The Mulliken charges of the Na atoms are between +0.75 and +0.83 and no significant overlap population with neighbouring atoms are observed, thus each atom shows according to the Zintl‐Klemm concept the expected charge of +1. For the isolated Bi1 the Mulliken charge is −1.04, which is also in line with the proposed charge. For the Sn atoms the Mulliken charges vary between −1.13 and +0.04. Here the charges are also in line with the specific connectivity of the atoms of the anionic substructure. Sn positions which are four‐fold bonded show charges close to 0 (the formal charge according to the Lewis formula is 0.) The three‐fold bonded Sn atoms show Mulliken charges of −0.45 to −0.64, which is close to the assumed formal charge of −1, and the two‐fold bonded Sn2 has the largest negative charge of −1.13, which would correspond to a formal charge of −2. This value is similar to one of the Bi1, which could hint, that the formal negative charge of the isolated Bi is actually more than −1.

Most interestingly, we found that for Na_13_Sn_24_Bi_3_ the overall Mulliken charge distribution is rather similar to Na_13_Sn_26_Bi. Although a charge of −3 for Bi1 is expected, almost the same Mulliken charge, as for Na_13_Sn_26_Bi is calculated, with a value of −1.05. Considering that Na_13_Sn_24_Bi_3_ possesses two more electrons than Na_13_Sn_26_Bi, the excess of charge is rather distributed over all Sn and Bi atoms of the network, than located at the Bi1 position.

Based on the study of the two borderline models Na_13_Sn_26_Bi and Na_13_Sn_24_Bi_3_ the electronic structure of the composition Na_13_Sn_25.73(2)_Bi_1.27(2)_ as determined by a single crystal structure refinement, reflects most probably a snapshot of the phase with a certain phase width Na_13_Sn_26–x_Bi_1+x_ with x ∈ [0;2]. Surprisingly, and in contrast to a simple rigid band filling model ‐ i. e. considering the band structure of Na_13_Sn_26_Bi and simply adding two electrons resulting in a metallic behaviour – both compounds are semiconductors even though Na_13_Sn_24_Bi_3_ is more electron rich. The Bi1 atom, which has no strong interactions with the surrounding atoms, therefore serves as a guest atom and electron buffer.

Thus, the reported compound is an intriguing example of a Zintl phase which can retain the semiconducting property despite being non‐electron precise with respect to the 8‐N rule. We conducted many experiments to vary the composition, but always found the approximate composition Na_13_Sn_25.73_Bi_1.27_. Powder diffractograms and single crystals show, within the standard deviations, always the same lattice parameters. We explain this with the compound's host‐guest nature, in which the guest atom Bi can slightly change the oxidation state without changing the stiff Sn network. The band structure is remarkably flexible in shifting bands at the Fermi level retaining the band gap.

### Comparison with the Electronic Structure of Na_5_Sn_13_ and Na_7_Sn_12_

As a starting point for this investigation we used the compound Na_5_Sn_13_, which has been regarded as a Zintl phase if that one very long Sn−Sn contact of 3.6  Å is considered as a two‐electron‐two center bond.^[11]^ Therefore, we calculated the band structure using the same computational methods for Na_5_Sn_13_ and also for the related Na_7_Sn_12_, which is known as an electron precise Zintl phase.

**Figure 7:**
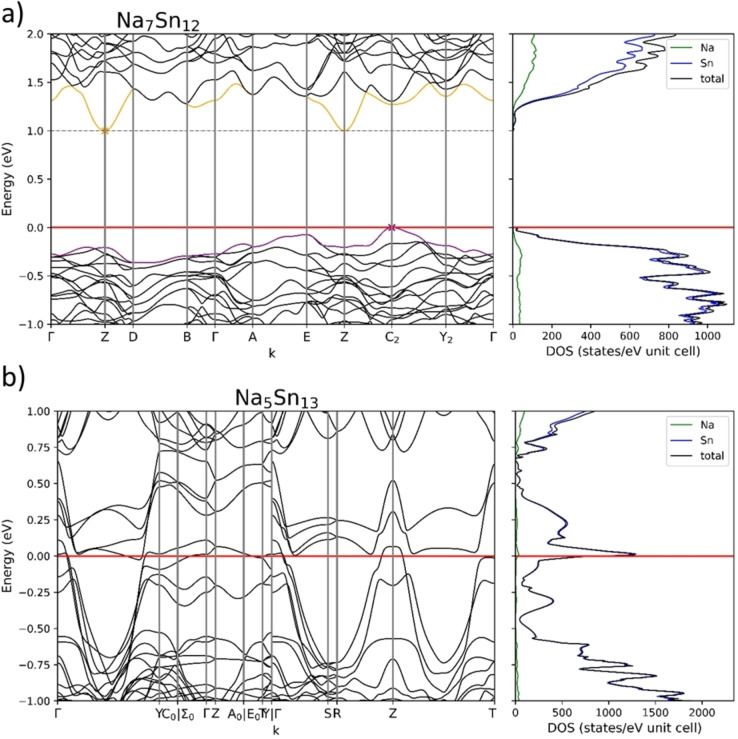
Band structure and density of states for a) Na_7_Sn_12_ with a band gap of 1.0 eV and b) metallic Na_5_Sn_13_.

According to the band structure shown in Figure 7, Na_7_Sn_12_ is a semiconductor with an indirect band gap of 1.0 eV and Na_5_Sn_13_ a metal. Na_7_Sn_12_ has, similar to Na_13_Sn_26_Bi, flat bands around the band gap with a low dispersion except for the lowest conduction band which also shows a large dispersion of around 0.4 eV. Focusing on the band structure of Na_5_Sn_13_^,^ which similar to Na_13_Sn_26_Bi and Na_13_Sn_24_Bi_3_ is not an electron precise compound at first glance, the band structure shows a large band dispersion and a high density of states at E_F_ as it is expected for a good metal. The partial DOS of the Sn11 atoms (Figure S4, Supp. Info), which have been reported to form an exceptionally long covalent bond,^[11]^ contribute significantly to the total DOS at E_F_ and support the hypothesis that they are also largely responsible for the metallic property of Na_5_Sn_13_.

Whereas in the ternary phases, the Sn versus Bi substitution opens a band gap, Na_5_Sn_13_ is a metal. Thus, introducing atoms with different size and different electronegativity values seems to be able to change the electronic property from conducting to semi‐conducting.

Another difference is observed with respect to the Na contributions in the density of states. The DOS of both compounds mostly consists of Sn states. For Na_5_Sn_3_ there is a small maximum in Na states at the Fermi‐Level, while this is absent in the Na_7_Sn_12_ Na DOS. The same holds for the modelled phases for Na_13_Sn_25.73_Bi_1.27_ where Na has only some small contribution around the Fermi level. This as well as the width of the band gap could hint, that the title compound is electronically closer related to the electron precise Zintl phase Na_7_Sn_12_ than Na_5_Sn_13_.

## Conclusions

Na_13_Sn_25.73_Bi_1.27_ represents the first ternary compound in the Na‐Sn‐Bi system. With respect to composition and structural details it is closely related to Na_5_Sn_13_. Both phases are exceptions to the Zintl‐Klemm concept since for the metallic Na_5_Sn_13_ unrealistically long covalent bonds of 3.16 Å are anticipated, whereas the same characteristics (non‐charge balanced and long Bi−Sn bonds) can be found in Na_13_Sn_25.73_Bi_1.27_ which surprisingly is a semiconductor with a band gap of 0.5 eV. The electronic situation was traced back to a loosely bound Bi atom–beside a second Bi atom that is part of the covalent Sn substructure–which acts as a guest atom according to Bi_x_@Na_13_Bi_y_Sn_27‐x‐y_ (x=1, y=0.27) and is capable to change its oxidation state and thus to uptake additional electrons allowing the system to be a semiconductor. Therefore, Na_13_Sn_25.73_Bi_1.27_ can be understood as a rare example of an open framework structure of Sn atoms comprising Bi atoms in the cavities. Sn atoms that form clathrate‐type framework structures based on K and Sn atoms are well known (K_8_Sn_44_ ^[23]^ or K_6_Sn_23_Bi_2_ ^[13]^). Open framework structures have also been described for examples in which Na atoms are located in cavities of a Sn−Zn framework.^[24]^ Na_5_Sn_13_ and Na_13_Sn_25.73_Bi_1.27_ are two additional examples showing the fantastic possibilities, with respect to chemical bonding and properties, of Zintl phases that are close but not perfect electron precise semiconductors.

## Experimental Section

### Synthesis

A Sample of the nominal composition ‘Na_5_Sn_12_Bi_1_’ was obtained from a mixture of elements Na : Sn:Bi with the composition 5 : 12 : 1. The mixture was heated at the rate of 120 °C/h to 650 °C, kept there for 12 h, then cooled at the rate of 120 °C/h to 270 °C where it is annealed for at least 5 days and, subsequently cooled to room temperature by turning off the furnace. The product is air and moisture sensitive and crystallized as irregular silvery crystals. Most of the crystals appear to be twinned (multiple twinning) and not suitable for single crystal diffraction. The X‐ray powder diagram of the product compares very well with the theoretical diagram calculated from the structure solution crystal data however, with some unindexed peaks indicating (an) additional yet unknown phase(s). The presence of the three elements Sn, Na and Bi was confirmed by EDX analysis, and no eventual contaminant was detected.

For single crystal growth the a sample of the nominal composition ‘Na_5_Sn_12_Bi_2_’ was synthesized from the elements in Ta ampoule using a two‐step temperature program: (1) heating up to 650 °C with the rate of 120 °C/h and holding the temperature for 12 hours and (2) slowly cooling down to 270 °C (rate 6 °C/h) and dwelling the sample for 240 hours. The sample was cooled down to room temperature by switching off the furnace.

### Powder X‐Ray Diffraction

For the powder X‐ray diffraction (PXRD) analysis, obtained samples were finely ground in an agate mortar, sealed in a glass capillary (inner diameter 0.3 mm, wall thickness 0.01 mm, Hilgenberg GmbH) using capillary wax (Hampton Research) and measured at room temperature using a STOE Stadi P powder diffractometer with Mo−Kα (λ=0.70932 Å) radiation, Ge (111) monochromator and a position sensitive Dectris MYTHEN DCS 1 K solid‐state detector. STOE WinXPOW program package^[25]^ was used for phase analysis, indexing, and refining cell parameters for the obtained phases.

### Single Crystal X‐Ray Diffraction

Crystals suitable for single crystal X‐ray diffraction (SCXRD) analysis were selected under a microscope inside a glovebox and transferred into glass capillaries (inner diameter 0.1–0.3 mm, wall thickness 0.01 mm, Hilgenberg GmbH) using a glass filament dipped in perfluoropolyalkyl ether (Galden Perfluorniated Fluid LSD 230, Solvay Specialty Polymers, viscosity 1800 cSt). The capillaries were then sealed airtight using capillary wax (Hampton Research) and mounted onto a single crystal X‐ray diffractometer with Mo K_α_ radiation (λ=0.71073 Å). Single‐crystal intensity data were collected at room temperature or in cold N_2_ stream (150 K), using a Stoe Stadivari diffractometer equipped with a micro focus GeniX 3D source (high flux Mo Kα radiation) and a DECTRIS PILATUS 300 K detector. Corrections of the raw data for background, polarization, and Lorentz effects were applied. Due to a Gaussian‐shaped primary X‐ray beam profile, a scaling procedure within LANA was applied along with the numerical absorption correction using X‐Red^[26]^ and X‐Shape^[27]^ software. The starting atomic parameters were usually obtained by Direct Method with the SHELXS‐2014.^[28]^ The structure was refined using SHELXL‐2014 (full‐matrix least‐squares on Fo2) with anisotropic atomic displacement parameters for all atoms. To check the composition, the occupancy parameters were refined in separate least‐squares cycles. Crystallographic data and selected data and details of the structure refinement for Na_13_Sn_26_Bi are listed in Table 1. Deposition Number CSD‐2387128 contains the supplementary crystallographic data for this structure. These data are provided free of charge by the joint Cambridge Crystallographic Data Centre and Fachinformationszentrum Karlsruhe.

Presence of Bi, along with Na and Sn in the new compound were confirmed by EDX analysis (Table S3, SI). Significant deviation of the composition according to the EDX results is occurring due to oxidation of the crystal.

### Electronic Structure Calculations

The computational studies of Na_13_Sn_26_Bi and Na_13_Sn_24_Bi_3_ were performed using the CRYSTAL17 program package and hybrid density functional methods.[^29^, ^30^] A hybrid exchange‐correlation functional after Perdew, Burke, and Ernzerhof (DFT‐PBE0) was used,^[31]^ Localized, Gaussian‐Type triple ζ‐valence+polarization level basis sets were used for Sn und Bi and split valence+polarization level basis sets for Na. The basis sets were derived from the molecular Karlsruhe basis sets (further basis set details are in the supporting information).[^32^, ^33^, ^34^] For the evaluation of the Coulomb and exchange integrals (TOLINTEG), tight tolerance factors of 8, 8, 8, 8, 16 were used for all calculations. The reciprocal space of Na_13_Sn_26_Bi and Na_13_Sn_24_Bi_3_ were sampled with 6×6×4 Monkhorst‐Pack‐type *k*‐point grids. The starting geometries were taken from experimental data, and both the lattice parameters and atomic positions were fully optimized within the constraints imposed by the space symmetry. The optimized structures were confirmed to be true local minima by means of harmonic frequency calculations at the Γ‐point (only very small imaginary frequency). Electronic band structures and density of states (DOS) were calculated. The Brillouin Zone paths of Γ‐X|Y‐Γ‐Z|R‐Γ‐T|U‐Γ‐V were provided by the web service *SeeK‐path*.^[35]^

## Author Contributions

Synthesis, powder diffraction and solution of the first single crystal was done by S.P. Synthesis and refinement of published single crystal data was performed by M. B. and V. H. helped with single crystal solution and refinement. Electronic structure calculations, data interpretation and writing of the manuscript was done by S. Z. Project administrator, manuscript editing, and review done by T. F. F..

## Conflict of Interests

The authors declare no conflict of interest.

1

## Supporting information

As a service to our authors and readers, this journal provides supporting information supplied by the authors. Such materials are peer reviewed and may be re‐organized for online delivery, but are not copy‐edited or typeset. Technical support issues arising from supporting information (other than missing files) should be addressed to the authors.

Supporting Information

## Data Availability

The data that support the findings of this study are available in the supplementary material of this article.

## References

[1] S. Ponou , N. Müller , T. F. Fässler , U. Häussermann , Inorg. Chem. 2005, 44, 7423. 16212368 10.1021/ic050603h

[2] E. Zintl , A. Harder , Z. Phys. Chem. (Berlin, Ger.) 1932, 16B, 206.

[3] T. F. Fässler , Z. anorg. allg. Chem. 2006, 632, 1125.

[4] Z. Du , R. A. Dunlap , M. N. Obrovac , J. Alloys Compd. 2014, 617, 271.

[5] F. Dubois , M. Schreyer , T. F. Fässler , Inorg. Chem. 2005, 44, 477. 15679372 10.1021/ic048770p

[6] T. F. Fässler , C. Kronseder , Angew. Chem. 1998, 110, 1641. 10.1002/(SICI)1521-3773(19980619)37:11<1571::AID-ANIE1571>3.0.CO;2-L 29710926

[7] Y. Grin , M. Baitinger , R. Kniep , H. G. von Schnering , Z. Kristallogr. - New Cryst. Struct. 1999, 214, 453.

[8] J. Mark , J. Wang , K. Wu , J. G. Lo , S. Lee , K. Kovnir , J. Am. Chem. Soc. 2019, 141, 11976. 31276390 10.1021/jacs.9b04653

[9] W. Müller , K. Volk , Z. Naturforsch., B: J. Chem. Sci. 1975, 30, 494.

[10] W. Müller , K. Volk , Z. Naturforsch., B: J. Chem. Sci. 1978, 33, 275.

[11] J. T. Vaughey , J. D. Corbett , Inorg. Chem. 1997, 36, 4316. 11670087 10.1021/ic9703181

[12] T. F. Fässler , S. Hoffmann , Inorg. Chem. 2003, 42, 5474. 12950190 10.1021/ic030148u

[13] T. F. Fässler , C. Kronseder , Z. anorg. allg. Chem. 1998, 624, 561.

[14] Z. Kristallogr , Cryst. Mater. 1991, 197, 269.

[15] J. Klein , B. Eisenmann , Z. Kristallogr. - Cryst. Mater. 1991, 196, 213.

[16] M. Asbrand , B. Eisenmann , Z. Naturforsch., B: J. Chem. Sci. 1993, 48, 452.

[17] B. Eisenmann , J. Klein , Z. Naturforsch., B: J. Chem. Sci. 1988, 43, 1156.

[18] B. Eisenmann , U. Rößler , Z. Kristallogr. - New Cryst. Struct. 1998, 213, 28.

[19] M. Asbrand , B. Eisenmann , J. Klein , Z. anorg. allg. Chem. 1995, 621, 576.

[20] S. Ponou, Germanides, germanide-tungstate double salts and substitution effects in Zintl phases. Dissertation, Universitätsbibliothek der TU München, München, **2006**.

[21] M. Boyko, Polar Intermetallics at the Border Between Hume-Rothery and Zintl Phases. Investigations in the Systems Alkali Metal–Tin with Late Transition and p-Block Metals. Dissertation, Universitätsbibliothek der TU München, München, **2019**.

[22] P. Pyykkö , M. Atsumi , Chem. - Eur. J. 2009, 15, 186. 19058281 10.1002/chem.200800987

[23] J. Gallmeier , H. Schäfer , A. Weiss , Z. Naturforsch., B: J. Chem. Sci. 1969, 24, 665.

[24] S. Stegmaier , S.-J. Kim , A. Henze , T. F. Fässler , J. Am. Chem. Soc. 2013, 135, 10654. 23692399 10.1021/ja401043b

[25] STOE & CIE GmbH, WinXPOW, Version 3.0.2.1 ed., STOE & Cie GmbH, Darmstadt, Germany, **2011**.

[26] X-RED32, STOE & Cie GmbH, Darmstadt, Germany **2016**.

[27] X-SHAPE, STOE & Cie GmbH,, Darmstadt, Germany, **2015**.

[28] G. M. Sheldrick, SHELXS-2014. *Program for the Determination of Crystal Structure*, University of Göttingen, Göttingen, Germany, **2014**.

[29] R. Dovesi, V. R. Saunders, C. Roetti, R. Orlando, C. M. Zicovich-Wilson, F. Pascale, B. Civalleri, K. Doll, N. M. Harrison, I. J. Bush, Crystal17, Turin, Italy, **2017**.

[30] R. Dovesi, A. Erba, R. Orlando, C. M. Zicovich-Wilson, B. Civalleri, L. Maschio, M. Rérat, S. Casassa, J. Baima, S. Salustro, et al., *Wiley Interdiscip. Rev.: Comput. Mol. Sci*. **2018**, *8*, e1360.

[31] J. P. Perdew , W. Yang , K. Burke , Z. Yang , E. K. U. Gross , M. Scheffler , G. E. Scuseria , T. M. Henderson , I. Y. Zhang , A. Ruzsinszky , et al., Proc. Natl. Acad. Sci. U. S. A 2017, 114, 2801. 28265085 10.1073/pnas.1621352114 PMC5358356

[32] A. J. Karttunen , T. F. Fässler , Chem. - Eur. J. 2014, 20, 6693. 24789147 10.1002/chem.201402251

[33] R. E. Stene , B. Scheibe , A. J. Karttunen , W. Petry , F. Kraus , Eur. J. Inorg. Chem. 2019, 2019, 3672.

[34] B. Scheibe , R. Haiges , S. I. Ivlev , A. J. Karttunen , U. Müller , K. O. Christe , F. Kraus , Eur. J. Inorg. Chem. 2020, 2020, 4483.

[35] Y. Hinuma , G. Pizzi , Y. Kumagai , F. Oba , I. Tanaka , Comput. Mater. Sci. 2017, 128, 140.

