# Supplementary material for: Open Sn Framework Structure Hosting Bi Guest atoms–Synthesis, Crystal and Electronic Structure of Na13Sn26Bi
Source: Chemistry. 2024 Nov 27;31(5):e202403592. doi: 10.1002/chem.202403592 (PMC11753384; doi:10.1002/chem.202403592)
Supplement: Supplementary file 1 — Supporting Information [file CHEM-31-e202403592-s001.pdf]

Chemistry–A European Journal

Supporting Information

Open Sn Framework Structure Hosting Bi Guest atoms–Synthesis, Crystal and Electronic Structure of $Na_{13}Sn_{26}Bi$

S. Zeitz, M. Boyko, S. Ponou, V. Hlukhyy, and T. F. Fässler*

Supporting Information

Open Sn Framework Structure Hosting Bi Guest atoms – Synthesis, Crystal and Electronic Structure of $Na_{13}Sn_{26}Bi$

S. Zeitz[a], M. Boyko[a], S. Ponou[a], V. Hlukky[a], T.F. Fässler[a]

Author Affiliation:

[a] School of Natural Science, Technical University of Munich, Chair of Inorganic Chemistry with Focus on Novel Materials, Lichtenbergstraße 4, D-85747 Garching, Germany.
Email: thomas.faessler@lrz.tum.de

1. Na-Sn-Pn (Pn = P, As, Sb) system: overview of known compounds

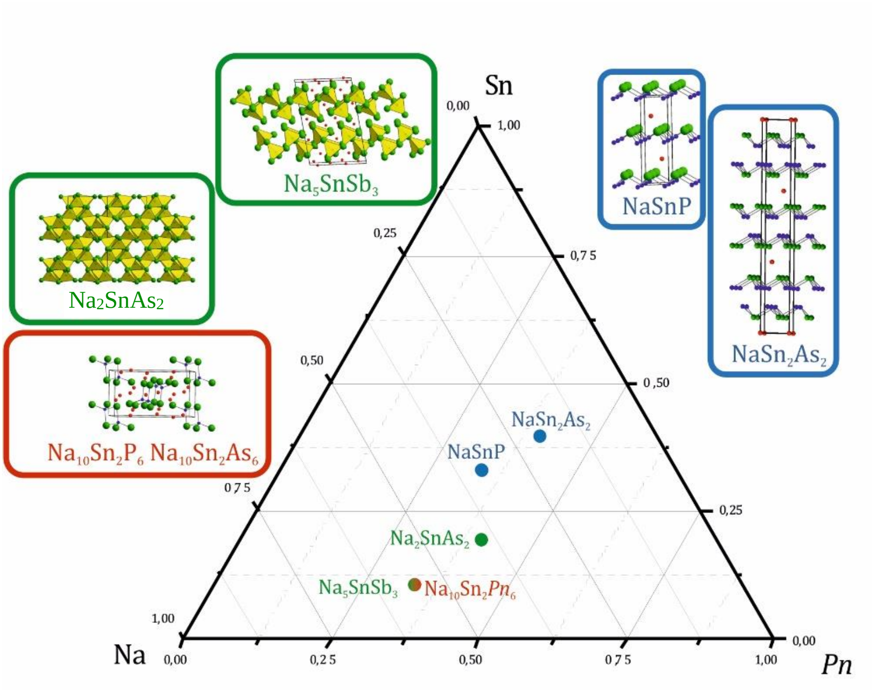

Figure S1: Crystal structures of the compounds in the Na-Sn-*Pn* (*Pn* = P, As, Sb) ternary systems. In red rectangles are phases with isolated fragments, blue – with two-dimensional nets, green – three-dimensional networks built by tetrahedrally coordinated Sn atoms. [3–8]

2. Additional information on the Crystal Structure:

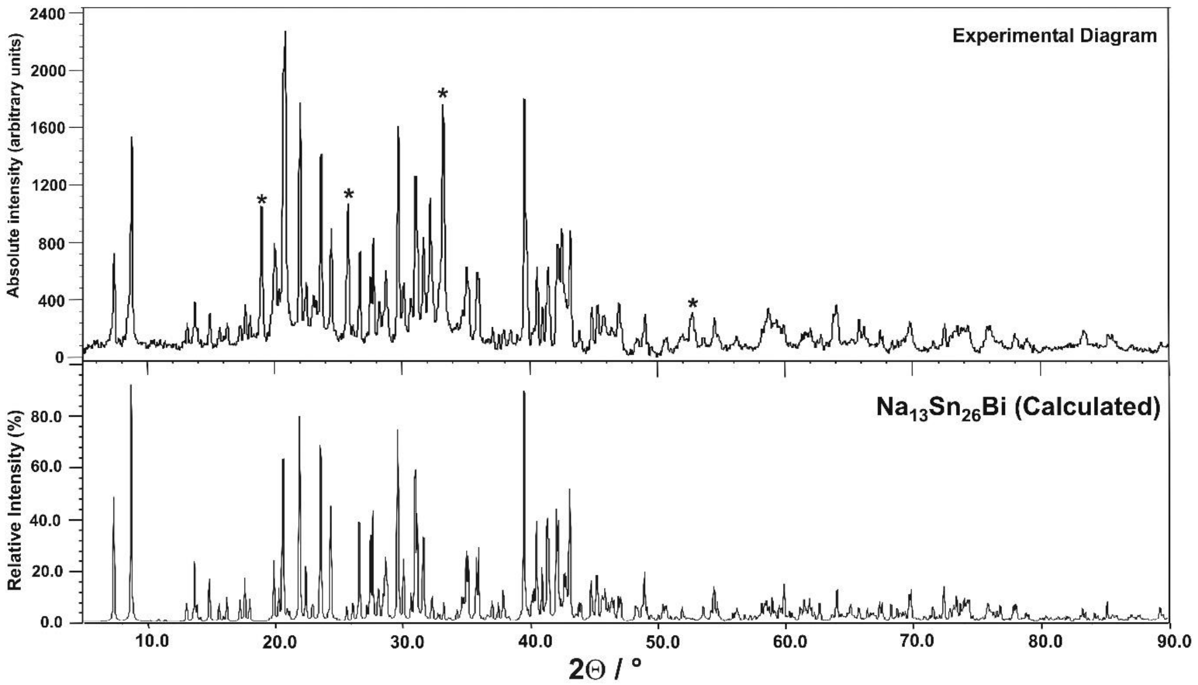

Figure S2. Experimental powder diagram of the reaction product Na:Sn:Bi = 5:12:1 (top) and theoretical powder diagram calculated for $Na_{13}Sn_{26}Bi$ (bottom). Strongest un-indexed reflections are labeled with stars (*).

Table S1: Atom coordinates and equivalent isotropic displacement parameters (Å^2) for the compound $Na_{13}Sn_{25.73(2)}Bi_{1.27(2)}$, E = statistical mixture of Sn and Bi.

Atom	Wyck.	S.O.F.	x	y	z	U_{eq}
Sn1	2i	1	0.44093(9)	0.43226(8)	0.0090(2)	0.0162(9)
Bi1	2i	0.5	0.5118(1)	0.0132(6)	0.0162(9)	0.0090(2)
E2	2i	0.864/0.136(8)	0.36986(8)	0.18593(7)	0.0095(3)	0.0095(3)
Sn3	2i	1	0.5403(1)	0.20270(9)	0.45310(7)	0.0080(2)
Sn4	2i	1	0.2026(1)	0.85338(9)	0.43784(8)	0.0091(2)
Sn5	2i	1	0.0342(1)	0.19197(9)	0.45582(7)	0.0079(2)
Sn6	2i	1	0.3007(1)	0.13760(9)	0.55179(8)	0.0086(2)
Sn7	2i	1	0.5875(1)	0.45092(9)	0.42016(8)	0.0101(2)
Sn8	2i	1	0.1261(1)	0.79320(9)	0.20159(8)	0.0097(2)
Sn9	2i	1	0.5746(1)	0.02976(9)	0.76238(7)	0.0083(2)
Sn10	2i	1	0.9075(1)	0.07131(9)	0.06880(8)	0.0087(2)
Sn11	2i	1	0.3719(1)	0.19887(9)	0.78769(8)	0.0090(2)
Sn12	2i	1	0.0706(1)	0.03159(9)	0.76016(7)	0.0082(2)
Sn13	2i	1	0.4097(1)	0.07286(9)	0.06582(8)	0.0094(2)
Na1	2i	1	0.3649(8)	0.5802(7)	0.2658(6)	0.031(2)
Na2	2i	1	0.2322(7)	0.2782(6)	0.0180(5)	0.021(1)
Na3	2i	1	0.2555(7)	0.2194(7)	0.2680(5)	0.018(1)
Na4	2i	1	0.6676(7)	0.2748(6)	0.0160(5)	0.018(1)
Na5	2i	1	0.7390(7)	0.2216(7)	0.2687(5)	0.019(1)
Na6	2i	1	0.1972(9)	0.4216(7)	0.7357(6)	0.032(2)
Na7	1c	1	0	1/2	0	0.037(3)

Table S2: Anisotropic displacement parameters ($Å^2$) for the compound $Na_{13}Sn_{25.73(2)}Bi_{1.27(2)}$.

**Atom**	U_{11}	U_{22}	U_{33}	U_{12}	U_{13}	U_{23}
Bi1	0.0171(2)	0.020(3)	0.020(3)	0.015(2)	0.008(2)	0.008(2)
Sn1	0.0077(4)	0.0081(4)	0.0109(4)	0.0037(3)	0.0025(3)	0.0021(3)
E2	0.0084(4)	0.0104(5)	0.0112(5)	0.0059(3)	0.0028(3)	0.0028(3)
Sn3	0.0076(4)	0.0084(4)	0.0080(4)	0.0035(3)	0.0021(3)	0.0023(3)
Sn4	0.0084(4)	0.0123(4)	0.0100(4)	0.0070(4)	0.0035(3)	0.0044(3)
Sn5	0.0079(4)	0.0080(4)	0.0080(4)	0.0038(3)	0.0023(3)	0.0021(3)
Sn6	0.0075(4)	0.0115(4)	0.0086(4)	0.0054(3)	0.0029(3)	0.0034(3)
Sn7	0.0093(4)	0.0091(4)	0.0120(4)	0.0051(4)	0.0024(3)	0.0021(3)
Sn8	0.0094(4)	0.0100(4)	0.0095(4)	0.0035(3)	0.0030(3)	0.0033(3)
Sn9	0.0083(4)	0.0094(4)	0.0064(4)	0.0026(3)	0.0019(3)	0.0026(3)
Sn10	0.0080(4)	0.0092(4)	0.0093(4)	0.0043(3)	0.0025(3)	0.0026(3)
Sn11	0.0092(4)	0.0096(4)	0.0091(4)	0.0048(3)	0.0028(3)	0.0029(3)
Sn12	0.0083(4)	0.0087(4)	0.0066(4)	0.0026(3)	0.0014(3)	0.0022(3)
Sn13	0.0085(4)	0.0102(4)	0.0090(4)	0.0041(3)	0.0019(3)	0.0021(3)
Na1	0.026(4)	0.032(4)	0.021(3)	0.003(3)	0.001(3)	0.004(3)
Na2	0.022(3)	0.019(3)	0.025(3)	0.011(3)	0.009(3)	0.006(3)
Na3	0.010(3)	0.036(4)	0.018(3)	0.015(3)	0.008(2)	0.014(3)
Na4	0.017(3)	0.016(3)	0.022(3)	0.013(3)	-0.001(2)	0.003(2)
Na5	0.010(3)	0.034(4)	0.017(3)	0.015(3)	0.008(2)	0.007(3)
Na6	0.033(4)	0.031(4)	0.025(4)	0.002(3)	0.010(3)	0.011(3)
Na7	0.072(8)	0.017(5)	0.007(4)	-0.001(4)	-0.005(5)	0.004(5)
Bi1	0.0171(2)	0.020(3)	0.020(3)	0.015(2)	0.008(2)	0.008(2)
Sn1	0.0077(4)	0.0081(4)	0.0109(4)	0.0037(3)	0.0025(3)	0.0021(3)
Sn2	0.0084(4)	0.0104(5)	0.0112(5)	0.0059(3)	0.0028(3)	0.0028(3)
Bi2	0.0084(4)	0.0104(5)	0.0112(5)	0.0059(3)	0.0028(3)	0.0028(3)
Sn3	0.0076(4)	0.0084(4)	0.0080(4)	0.0035(3)	0.0021(3)	0.0023(3)
Sn4	0.0084(4)	0.0123(4)	0.0100(4)	0.0070(4)	0.0035(3)	0.0044(3)
Sn5	0.0079(4)	0.0080(4)	0.0080(4)	0.0038(3)	0.0023(3)	0.0021(3)
Sn6	0.0075(4)	0.0115(4)	0.0086(4)	0.0054(3)	0.0029(3)	0.0034(3)
Sn7	0.0093(4)	0.0091(4)	0.0120(4)	0.0051(4)	0.0024(3)	0.0021(3)
Sn8	0.0094(4)	0.0100(4)	0.0095(4)	0.0035(3)	0.0030(3)	0.0033(3)
Sn9	0.0083(4)	0.0094(4)	0.0064(4)	0.0026(3)	0.0019(3)	0.0026(3)
Sn10	0.0080(4)	0.0092(4)	0.0093(4)	0.0043(3)	0.0025(3)	0.0026(3)
Sn11	0.0092(4)	0.0096(4)	0.0091(4)	0.0048(3)	0.0028(3)	0.0029(3)
Sn12	0.0083(4)	0.0087(4)	0.0066(4)	0.0026(3)	0.0014(3)	0.0022(3)
Sn13	0.0085(4)	0.0102(4)	0.0090(4)	0.0041(3)	0.0019(3)	0.0021(3)

Table S3: Results of the EDX analysis of the crystal with the refined composition $Na_{13}Sn_{25.73}Bi_{1.27}$ from the sample '$Na_5Sn_{12}Bi_2$'.

	**Na (at. %)**	**Sn (at. %)**	**Bi (at. %)**
EDX	39(12)	46(6)	15(6)
$Na_{13}Sn_{25.73}Bi_{1.27}$	32.5	64.3	3.2

Table S4: Interatomic distances in the $Na_{13}Sn_{25.73(2)}Bi_{1.27(2)}$.

**Atom types**		**Distance (Å)**	**Atom types**		**Distance (Å)**
		**Sn-Sn, Sn-Bi, Bi-Bi**			
Bi1	Sn11	3.145(8)	Sn1	Sn7	2.889(1)
E2	Sn1	2.965(1)		Sn5	2.892(2)
	Sn10	2.985(1)		Sn1	2.911(1)
Sn4	Sn5	2.829(2)		E2	2.965(1)
	Sn3	2.837(2)	Sn3	Sn6	2.835(2)
	Sn6	2.843(2)		Sn4	2.837(2)
	Sn8	2.861(2)		Sn9	2.889(1)
Sn6	Sn5	2.822(1)		Sn7	2.947(2)
	Sn3	2.835(2)	Sn5	Sn6	2.822(1)
	Sn4	2.843(1)		Sn4	2.829(2)
	Sn11	2.854(2)		Sn12	2.848(1)
Sn8	Sn4	2.861(2)		Sn1	2.892(2)
	Sn12	2.878(2)	Sn7	Sn7	2.879(1)
	Sn9	2.880(1)		Sn1	2.889(1)
Sn10	Sn13	2.887(1)		Sn3	2.947(2)
	Sn12	2.900(2)	Sn9	Sn11	2.870(2)
	Sn10	2.936(1)		Sn8	2.880(1)
	E2	2.985(1)		Sn3	2.890(1)
Sn12	Sn5	2.848(1)		Sn13	2.907(2)
	Sn11	2.876(1)	Sn11	Sn6	2.854(2)
	Sn8	2.878(2)		Sn9	2.870(2)
	Sn10	2.900(2)		Sn12	2.877(1)
				Bi1	3.145(8)
			Sn13	Sn13	2.880(1)
				Sn10	2.887(1)
				Sn9	2.907(2)
		**Na-Sn, Na-Bi, Na-Na**			
Na1	E2	3.298(7)	Na2	Bi1	3.20(1)
	Sn3	3.431(7)		E2	3.243(7)
	Sn11	3.457(8)		Sn13	3.250(8)
	Sn7	3.516(9)		Bi1	3.27(1)
	Na2	3.521(8)		Sn12	3.324(5)
	Sn6	3.614(6)		Sn11	3.377(7)
	Bi1	3.65(1)		Na1	3.521(8)
	Sn8	3.759(9)		Sn10	3.540(6)
	Sn4	3.804(8)		Na3	3.59(1)
	Bi1	3.89 (1)		Na7	3.618(7)
	Na6	3.99 (1)		Sn8	3.696(6)
	Na3	4.00(1)		Sn10	3.703(7)
Na3	Sn13	3.271(7)		Na4	3.967(9)
	Sn7	3.288(5)	Na4	Bi1	3.22(3)
	E2	3.302(8)		E2	3.246(6)
	Sn3	3.332(7)		Sn13	3.262(7)
	Sn1	3.365(7)		Sn9	3.274(5)
	Sn9	3.387(8)		Bi1	3.30(2)
	Sn10	3.389(5)		Sn11	3.384(6)
	Sn5	3.513(7)		Sn13	3.527(7)
	Sn12	3.524(7)		Na6	3.534(8)

	Na2	3.59(1)		Na5	3.59(1)
	Na1	4.00(1)		Sn8	3.623 (7)
Na5	Sn13	3.295(5)		Sn10	3.624(8)
	E2	3.298(7)		Na7	3.631(7)
	Sn3	3.315(7)		Na2	3.967(9)
	Sn7	3.319(7)	Na6	E2	3.298(9)
	Sn10	3.351(7)		Sn11	3.430(9)
	Sn9	3.439(7)		Sn5	3.463(6)
	Sn1	3.443(5)		Na4	3.534(8)
	Sn5	3.484(7)		Sn7	3.554(9)
	Sn12	3.550(8)		Sn6	3.623(8)
	Na4	3.59(1)		Bi1	3.62(1)
	Na6	3.97(1)		Sn8	3.833(9)
Na7	2× Sn8	3.186(8)		Bi1	3.86(1)
	2× E2	3.300(1)		Sn4	3.925(6)
	2× Na2	3.618(7)		Na5	3.97(1)
	2× Na4	3.631(7)		Na1	3.98(1)
	2× Na1	4.076(7)		Sn1	4.058(9)
	2× Na6	4.080(9)		Na7	4.080(9)

3. Electronic Band Structure Calculations:

Detailed description of the used basis sets and basis set listings in CRYSTAL format.

Na: taken from literature[1].

```
11 5
0 0 5 2.0 1.0
  4098.2003908     -.58535911879E-02
  616.49374031     -.43647161872E-01
  139.96644001     -.19431465884
  39.073441051     -.48685065731
  11.929847205     -.41881705137
0 0 3 2.0 1.0
  20.659966030      .85949689854E-01
  1.9838860978     -.56359144041
  .64836323942     -.51954009048
0 0 1 1.0 1.0
  0.32              1.0000000000
0 1 1 0.0 1.0
  0.16              1.0 1.0
0 2 5 6.0 1.0
  75.401862017     .154353625324E-01
  17.274818978     .997382931840E-01
  5.1842347425     .312095939659
  1.6601211973     .492956748074
  .51232528958     .324203983180
```

Sn: taken from literature[2].

```
250 13
INPUT
22   0   2   4   4   2   0
17.4204140000000  279.988682000000  0
7.63115500000000  62.3778100000000  0
16.1310240000000  66.1625230000000  0
15.6280770000000  132.174396000000  0
7.32560800000000  16.3394170000000  0
6.94251900000000  32.4889590000000  0
15.5149760000000  36.3874410000000  0
15.1881600000000  54.5078410000000  0
5.45602400000000  8.69682300000000  0
5.36310500000000  12.8402080000000  0
12.2823480000000 -12.5763330000000  0
12.2721500000000 -16.5959440000000  0
0 0 4 2.0 1.0
  1577.0715931      0.17042767713E-03
  235.26601078      0.81467057272E-03
  38.206330645     -0.39057904293E-02
  13.097031765      0.53245922343
0 0 2 2.0 1.0
  11.673161352       1.5435287275
  5.9463871497      0.76421510041
0 0 1 0.0 1.0
 1.8924497043       1.0000000000
0 0 1 0.0 1.0
 0.88429486443       1.0000000000
0 0 1 0.0 1.0
 0.23543697233       1.0000000000
0 1 1 0.0 1.0
 0.09                1.0 1.0
0 2 3 6.0 1.0
  221.55767496      0.31125177983E-03
  21.084021433      0.31108097016E-01
  8.7600138521     -0.27571560918
0 2 3 2.0 1.0
  2.5902185134      0.45912328666
  1.3409064118      0.49682867217
 0.67241607517      0.18962377821
0 2 1 0.0 1.0
  0.27895803477     1.0000000000
0 3 6 10.0 1.0
  108.33210154      0.46561853277E-03
  23.703936630      0.54063163067E-01
  22.339843906     -0.58928768877E-01
  4.0874834028      0.19588500896
  1.9737354146      0.42301799185
 0.90158257692      0.39252716176
0 3 1 0.0 1.0
 0.38237153649      1.0000000000
0 3 1 0.0 1.0
 0.191185768245     1.0000000000
0 4 1 0.0 1.0
 0.293928485        1.0000000000
```

Bi: taken from literature[9].

```
283 13
INPUT
23. 0 2 4 4 2 2
13.043090 283.264227 0
8.221682   62.471959 0
10.467777  72.001499 0
9.118901  144.002277 0
6.754791    5.007945 0
6.252592    9.991550 0
8.081474   36.396259 0
7.890595   54.597664 0
4.955556    9.984294 0
4.704559   14.981485 0
4.214546   13.713383 0
4.133400   18.194308 0
6.205709  -10.247443 0
6.227782  -12.955710 0
0 0 4 2.0 1.0
  716.41435310      0.31254307133E-03
  83.806059047      0.17624768946E-02
  21.116962853     -0.21910983437
  15.491448187      0.40411224931
0 0 2 2.0 1.0
  23.213322651     -0.68255758685E-01
  6.6412776932      0.97888046471
0 0 1 0.0 1.0
 1.7668753097       1.0000000000
0 0 1 0.0 1.0
 0.87636753866      1.0000000000
0 0 1 0.0 1.0
 0.26185055933      1.0000000000
0 1 1 0.0 1.0
 0.10               1.0 1.0
0 2 3 6.0 1.0
  15.249644669      0.74560356000
  14.846176053     -0.85578637338
  7.0636826784      0.40149159592
0 2 3 3.0 1.0
  2.5802708340      0.35542729633
  1.4990870077      0.63976991890
 0.75411119473      0.32332773839
0 2 1 0.0 1.0
  0.29617825843     1.0000000000
0 3 6 10.0 1.0
  66.404481948      0.38102878348E-03
  13.858426961      0.10746152442E-01
  7.0654519000     -0.71947646845E-01
  2.5252144035      0.26195974989
  1.3419585000      0.42594750000
 0.68340941000      0.33680325627
0 3 1 0.0 1.0
 0.32934755420      1.0000000000
0 3 1 0.0 1.0
 0.1646737771     1.0000000000
0 4 1 0.0 1.0
 0.31271            1.0000000000
```

Table S5: Comparison of the cell parameters between the experimentally determined and the optimized cell parameters of the two stoichiometric models.

	Experiment [Å]	$Na_{13}Sn_{26}Bi$ [Å]	Δ(Exp.-Calc.) [%]	$Na_{13}Sn_{24}Bi_3$ [Å]	Δ(Exp.-Calc.) [%]
a	9.0826	9.0571	-0.28	9.0449	-0.42
b	11.2527	11.2931	0.36	11.1327	-1.08
c	13.2278	13.2160	-0.09	13.4630	1.75
α	112.114	112.7620	0.57	110.5816	-1.39
β	99.818	99.7086	-0.11	100.5517	0.73
γ	101.379	101.2220	-0.16	101.7537	0.37

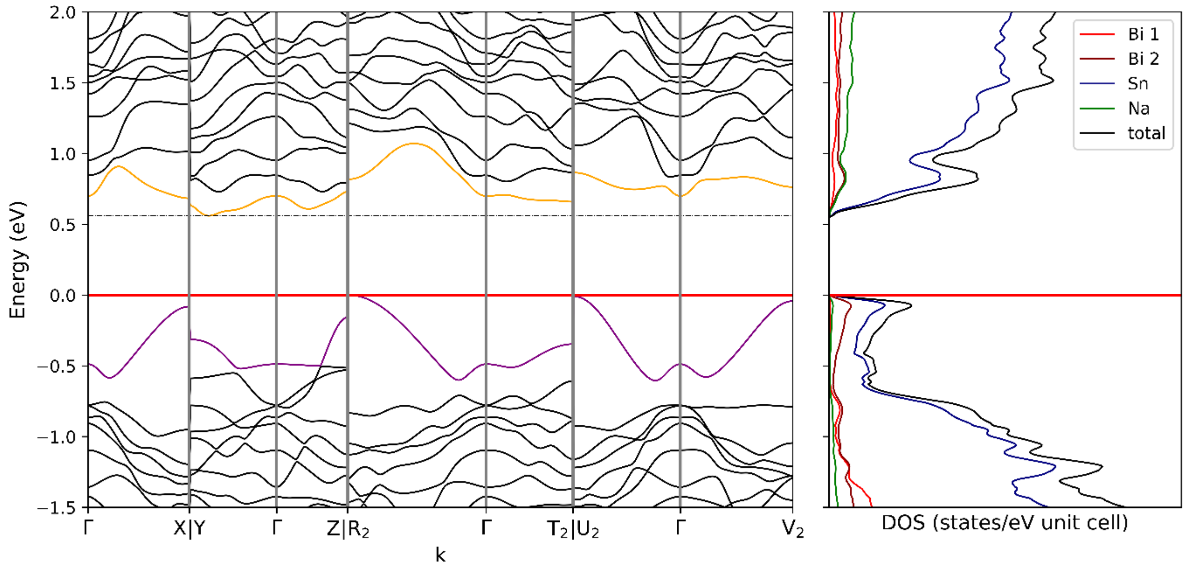

Figure S3: Band structure of $Na_{13}Sn_{24}Bi_3$ with atomic position resolved DOS for Bi.

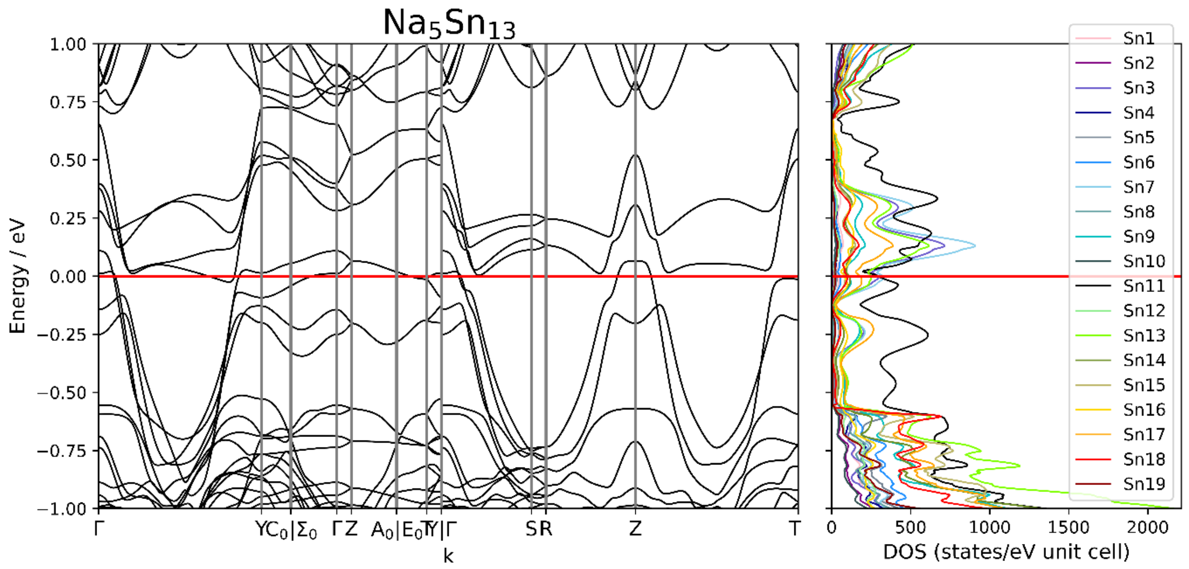

Figure S4: Band structure of Na_4Sn_{13} with atomic positions resolved DOS for Sn

Table S6: Partial charges from the Mullikan analysis. The effective charge Z_{eff} was subtracted from the number of electrons of the neutral element to get the partial charge.

$Na_{13}Sn_{26}Bi$				$Na_{13}Sn_{24}Bi_3$			
Atom	Z_{eff}:	part. ch.:	ass. ch.:	Atom	Z_{eff}:	part. ch.:	ass. ch.:
Bi1	24.038	-1.038	-1	Bi1	24.053	-1.053	-1
Sn1	22.167	-0.167	0	Sn1	22.247	-0.247	0
Sn2	23.132	-1.132	-2	Bi2	24.023	-1.023	-1
Sn3	22.348	-0.348	0	Sn3	22.310	-0.310	0
Sn4	21.96	0.04	0	Sn4	21.968	0.032	0
Sn5	22.236	-0.236	0	Sn5	22.298	-0.298	0
Sn6	22.029	-0.029	0	Sn6	22.015	-0.015	0
Sn7	22.535	-0.535	-1	Sn7	22.559	-0.559	-1
Sn8	22.538	-0.538	-1	Sn8	22.549	-0.549	-1
Sn9	22.168	-0.168	0	Sn9	22.152	-0.152	0
Sn10	22.26	-0.26	0	Sn10	22.185	-0.185	0
Sn11	22.453	-0.453	-1	Sn11	22.485	-0.485	-1
Sn12	22.148	-0.148	0	Sn12	22.15	-0.150	0
Sn13	22.64	-0.64	-1	Sn13	22.665	-0.665	-1
Na1	10.217	0.783	1	Na1	10.21	0.790	1
Na2	10.236	0.764	1	Na2	10.234	0.766	1
Na3	10.169	0.831	1	Na3	10.177	0.823	1
Na4	10.229	0.771	1	Na4	10.228	0.772	1
Na5	10.17	0.83	1	Na5	10.175	0.825	1
Na6	10.222	0.778	1	Na6	10.213	0.787	1
Na7	10.246	0.754	1	Na7	10.259	0.741	1

Table S7: Overlap population between the first six nearest neighbours calculated via Mullikan analysis for $Na_{13}Sn_{24}Bi_3$.

atom A	atom B	R(A-B) [Å]	overlap Population (AB)	atom A	atom B	R(A-B) [Å]	overlap Population (AB)
Bi1	Na2	3.118	0.047	Sn11	Sn6	2.885	0.24
	Na4	3.215	0.046		Sn12	2.889	0.234
	Sn11	3.317	0.098		Sn9	2.889	0.236
	Na1	3.904	0.019		Bi1	3.317	0.098
	Na6	3.913	0.02		Na4	3.358	0.013
	Na7	4.522	0.008		Na1	3.36	0.023
Sn1	Sn7	2.911	0.21	Sn12	Sn10	2.881	0.264
	Sn2	2.92	0.042		Sn11	2.889	0.234
	Sn5	2.934	0.217		Sn8	2.89	0.242
	Bi2	3.17	0.051		Sn10	2.912	0.255
	Na5	3.351	0.016		Na2	3.307	0.007
	Na3	3.507	0.009		Na3	3.52	0.009
Bi2	Sn10	2.952	0.206	Sn13	Sn10	2.868	0.238
	Sn1	3.17	0.051		Sn13	2.924	0.203
	Na7	3.171	0.043		Sn9	2.929	0.231
	Na2	3.174	0.048		Na4	3.218	0.028
	Na4	3.215	0.042		Na5	3.259	0.024

	Na3	3.271	0.034		Na2	3.281	0.029
Sn3	Sn4	2.834	0.27	Na1	Bi2	3.355	0.037
	Sn6	2.843	0.264		Sn11	3.36	0.023
	Sn9	2.911	0.253		S7	3.414	0.032
	Sn7	2.951	0.215		Sn3	3.542	0.01
	Na3	3.284	0.012		Sn6	3.58	0.01
	Na5	3.343	0.012		Na2	3.681	0.003
Sn4	Sn3	2.834	0.27	Na2	Bi1	3.118	0.047
	Sn6	2.84	0.286		Bi2	3.174	0.048
	Sn5	2.848	0.26		Sn13	3.281	0.029
	Sn8	2.884	0.258		Sn12	3.307	0.007
	Na6	3.84	0.006		Sn10	3.381	0.007
	Na1	3.985	0.004		Sn11	3.386	0.013
Sn5	Sn6	2.835	0.259	Na3	Bi2	3.271	0.034
	Sn4	2.848	0.26		Sn3	3.284	0.012
	Sn12	2.881	0.264		Sn13	3.333	0.023
	Sn1	2.934	0.217		Sn7	3.358	0.019
	Na5	3.334	0.012		Sn9	3.412	0.009
	Na3	3.425	0.01		Sn5	3.425	0.01
Sn6	Sn5	2.835	0.259	Na4	Bi2	3.215	0.042
	Sn4	2.84	0.286		Bi1	3.215	0.046
	Sn3	2.843	0.264		Sn13	3.218	0.028
	Sn11	2.885	0.24		Sn9	3.261	0.009
	Na1	3.58	0.01		Sn11	3.358	0.013
	Na6	3.69	0.008		Sn13	3.499	0.009
Sn7	Sn7	2.896	0.221	Na5	Sn13	3.259	0.024
	Sn1	2.911	0.21		Sn5	3.334	0.012
	Sn3	2.951	0.215		Sn3	3.343	0.012
	Na3	3.358	0.019		Sn13	3.351	0.016
	Na5	3.391	0.021		Bi2	3.37	0.028
	Na1	3.414	0.032		Sn7	3.391	0.021
Sn8	Sn4	2.884	0.258	Na6	Sn11	3.437	0.021
	Sn12	2.89	0.242		Bi2	3.443	0.031
	Sn9	2.897	0.244		Sn7	3.505	0.032
	Na7	3.233	0.04		Sn5	3.539	0.009
	Na6	3.659	0.015		Sn8	3.659	0.015
	Na1	3.729	0.013		Sn6	3.69	0.008
Sn9	Sn11	2.889	0.236	Na7	Bi2	3.171	0.043
	Sn8	2.897	0.244		Sn8	3.233	0.04
	Sn3	2.911	0.253		Na4	3.606	0.002
	Sn13	2.929	0.231		Na2	3.613	0.003
	Na4	3.261	0.009		Na1	4.187	0.001
	Na3	3.412	0.009		Na6	4.205	0.001
Sn10	Sn13	2.868	0.238				
	Sn10	2.869	0.262				
	Sn12	2.912	0.255				
	Bi2	2.952	0.206				
	Na2	3.381	0.007				
	Na3	3.495	0.008				

Table S8: Overlap population between the first six nearest neighbours calculated via Mullikan analysis for $Na_{13}Sn_{26}Bi$.

atom A	atom B	R(A-B) [Å]	overlap Population (AB)	atom A	atom B	R(A-B) [Å]	overlap Population (AB)
Bi1	Na2	3.232	0.043	Sn11	Sn6	2.862	0.246
	Sn11	3.247	0.084		Sn9	2.877	0.234
	Na4	3.268	0.043		Sn12	2.881	0.236
	Na6	3.654	0.029		Bi1	3.247	0.084
	Na1	3.705	0.027		Na2	3.353	0.014
	Na7	4.529	0.008		Na4	3.36	0.013
Sn1	Sn5	2.89	0.237	Sn12	Sn10	2.855	0.267
	Sn7	2.897	0.234		Sn8	2.878	0.246
	Sn1	2.907	0.266		Sn11	2.881	0.236
	Sn2	2.961	0.245		Sn10	2.906	0.251
	Na3	3.336	0.011		Na2	3.324	0.007
	Na5	3.419	0.01		Na3	3.502	0.009
Sn2	Sn1	2.961	0.245	Sn13	Sn13	2.887	0.21
	Sn10	2.983	0.21		Sn10	2.889	0.24
	Na2	3.229	0.046		Sn9	2.909	0.233
	Na4	3.234	0.043		Na2	3.221	0.033
	Na1	3.292	0.044		Na4	3.229	0.029
	N6	3.3	0.045		Na3	3.247	0.025
Sn3	Sn6	2.839	0.256	Na1	Sn2	3.292	0.044
	Sn4	2.843	0.261		Na2	3.43	0.005
	Sn9	2.892	0.25		Sn11	3.445	0.019
	Sn7	2.956	0.217		Sn3	3.453	0.012
	Na5	3.291	0.012		Sn7	3.527	0.026
	Na2	3.325	0.01		Sn6	3.609	0.009
Sn4	Sn5	2.828	0.273	Na2	Sn13	3.221	0.033
	Sn3	2.843	0.261		Sn2	3.229	0.046
	Sn6	2.843	0.284		Bi1	3.232	0.043
	Sn8	2.862	0.275		Sn12	3.324	0.007
	Na1	3.805	0.006		Sn11	3.353	0.014
	Na6	4.009	0.004		Na1	3.43	0.005
Sn5	Sn6	2.826	0.263	Na3	Sn13	3.247	0.025
	Sn4	2.828	0.273		Sn7	3.299	0.017
	Sn12	2.855	0.267		Sn2	3.315	0.027
	Sn1	2.89	0.237		Sn3	3.325	0.01
	Na5	3.468	0.009		S1	3.336	0.011
	Na6	3.494	0.012		Sn10	3.349	0.013
Sn6	Sn5	2.826	0.263	Na4	Sn13	3.229	0.029
	Sn3	2.839	0.256		Sn2	3.234	0.043
	Sn4	2.843	0.284		Sn9	3.252	0.007
	Sn11	2.862	0.246		Bi1	3.268	0.043
	Na1	3.609	0.009		Sn11	3.36	0.013
	Na6	3.611	0.01		Na6	3.443	0.005
Sn7	Sn7	2.891	0.195	Na5	Sn3	3.291	0.012

	Sn1	2.897	0.234		Sn13	3.292	0.02
	Sn3	2.956	0.217		Sn2	3.309	0.029
	Na3	3.299	0.017		Sn7	3.309	0.021
	Na5	3.309	0.021		Sn10	3.321	0.012
	Na1	3.527	0.026		Sn1	3.419	0.01
Sn8	Sn4	2.862	0.275	Na6	Sn2	3.3	0.045
	Sn12	2.878	0.246		Sn11	3.404	0.02
	Sn9	2.881	0.243		Na4	3.443	0.005
	Na7	3.145	0.044		Sn5	3.494	0.012
	Na4	3.622	0.013		Sn7	3.528	0.029
	Na2	3.726	0.012		Sn6	3.611	0.01
Sn9	Sn11	2.877	0.234	Sn7	Sn13	3.145	0.044
	Sn8	2.881	0.243		Sn2	3.313	0.034
	Sn3	2.892	0.25		Na2	3.64	0.002
	Sn13	2.909	0.233		Na4	3.667	0.002
	Na4	3.252	0.007		Na1	4.029	0.001
	Na3	3.374	0.01		Na6	4.055	0.001
Sn10	Sn13	2.889	0.24				
	Sn12	2.906	0.251				
	Sn10	2.928	0.257				
	Sn2	2.983	0.21				
	Na5	3.321	0.012				
	Na3	3.349	0.013				

References:

[1] R. E. Stene, B. Scheibe, A. J. Karttunen, W. Petry, F. Kraus, *Eur. J. Inorg. Chem.* **2019**, *2019*, 3672.

[2] A. J. Karttunen, T. F. Fässler, *Chemistry (Weinheim an der Bergstrasse, Germany)* **2014**, *20*, 6693.

[3] B. Eisenmann, U. Rößler, *Zeitschrift für Kristallographie - New Crystal Structures* **1998**, *213*, 28.

[4] J. Klein, B. Eisenmann, *Zeitschrift für Kristallographie - Crystalline Materials* **1991**, *196*.

[5] B. Eisenmann, J. Klein, *Zeitschrift für Naturforschung B* **1988**, *43*, 1156.

[6] M. Asbrand, B. Eisenmann, *Zeitschrift für Naturforschung B* **1993**, *48*, 452.

[7] M. Asbrand, B. Eisenmann, J. Klein, *Z. anorg. allg. Chem.* **1995**, *621*, 576.

[8] *Zeitschrift für Kristallographie - Crystalline Materials* **1991**, *197*, 269.

[9] B. Scheibe, R. Haiges, S. I. Ivlev, A. J. Karttunen, U. Müller, K. O. Christe, F. Kraus, *Eur. J. Inorg. Chem.* **2020**, *2020*, 4483.
